# Use of amplified *Mycobacterium tuberculosis* direct test in respiratory samples from HIV-infected patients in Brazil*

**DOI:** 10.1590/S1806-37132014000200008

**Published:** 2014

**Authors:** Leonardo Bruno Paz Ferreira Barreto, Maria Cristina da Silva Lourenço, Valéria Cavalcanti Rolla, Valdiléia Gonçalves Veloso, Gisele Huf

**Affiliations:** Oswaldo Cruz Foundation, Rio de Janeiro, Brazil; Oswaldo Cruz Foundation, Rio de Janeiro, Brazil; Oswaldo Cruz Foundation, Rio de Janeiro, Brazil; Oswaldo Cruz Foundation, Rio de Janeiro, Brazil; Oswaldo Cruz Foundation, Rio de Janeiro, Brazil

**Keywords:** Molecular diagnostic techniques, Tuberculosis, HIV, Molecular probe techniques

## Abstract

**OBJECTIVE:**

To compare the accuracy of the amplified *Mycobacterium tuberculosis* direct (AMTD) test with reference methods for the laboratory diagnosis of tuberculosis in HIV-infected patients.

**METHODS:**

This was a study of diagnostic accuracy comparing AMTD test results with those obtained by culture on Löwenstein-Jensen (LJ) medium and by the BACTEC Mycobacteria Growth Indicator Tube 960 (BACTEC MGIT 960) system in respiratory samples analyzed at the Bioassay and Bacteriology Laboratory of the Oswaldo Cruz Foundation Evandro Chagas Clinical Research Institute in the city of Rio de Janeiro, Brazil.

**RESULTS:**

We analyzed respiratory samples collected from 118 patients, of whom 88 (74.4%) were male. The mean age was 36.6 ± 10.6 years. Using the AMTD test, the BACTEC MGIT 960 system, and LJ culture, we identified *M. tuberculosis* complex in 31.0%, 29.7%, and 27.1% of the samples, respectively. In comparison with LJ culture, the AMTD test had a sensitivity, specificity, positive predictive value, and negative predictive value of 87.5%, 89.4%, 75.7%, and 95.0%, respectively, for LJ culture, whereas, in comparison with the BACTEC MGIT 960 system, it showed values of 88.6%, 92.4%, 83.8%, and 94.8%, respectively.

**CONCLUSIONS:**

The AMTD test showed good sensitivity and specificity in the population studied, enabling the laboratory detection of *M. tuberculosis* complex in paucibacillary respiratory specimens.

## Introduction

Even though more than a century has passed since the discovery of the etiologic agent of tuberculosis, i.e., *Mycobacterium tuberculosis*, the disease remains a public health problem worldwide. Each person with active tuberculosis will infect between 10 and 15 people every year. ^(^ ^1^ ^)^ It is estimated that at least one of every 10 people who have come in contact with the tuberculosis bacillus will develop the disease and that, in HIV-infected patients, this risk is 20 to 40 times higher.^(^ ^2^ ^)^ Studies evaluating survival in patients with tuberculosis/HIV co-infection have shown that the risk of death is higher in these patients than in HIV-infected patients without tuberculosis.^(^ ^3^ ^-^ ^6^ ^)^

Mycobacterial culture on solid Lowenstein-Jensen (LJ) medium is considered the gold standard isolation method.^(^ ^7^ ^)^ Although the limitation of this method is the long incubation period (2-8 weeks), it is used by most developing countries because of its low cost. Techniques such as nucleic acid amplification and automated liquid culture systems are costly and depend on sophisticated tools, which prevents their routine use in poor countries.

In the last decade, laboratory tests for detection of *M. tuberculosis* have evolved considerably.^(^ ^8^ ^)^ Today we have new methods, such as GeneXpert (Cepheid, Sunnyvale, CA, USA), which can yield results in 2 h, detecting *M. tuberculosis* complex and determining whether the strains are rifampin resistant; however, this method remains costly and has just begun to be used and validated for use in Brazil. The amplified* Mycobacterium tuberculosis* direct (AMTD) test (Gen-Probe, San Diego, CA, USA) can detect *M. tuberculosis* complex rRNA in approximately 3 h. This test was approved by the Food and Drug Administration for use in smear microscopy-positive respiratory samples in 1995, and, after it was improved in 1999, it was approved for use in smear microscopy-negative samples.^(^ ^9^ ^)^ There is still need for a better understanding of the performance of this test for paucibacillary patients, such as HIV-infected patients in Brazil, since the quality of their samples makes it difficult to establish a laboratory diagnosis, even by gold standard methods, such as liquid culture. The objective of the present study was to compare the diagnostic accuracy of the AMTD test with other culture methods in respiratory samples collected from HIV-infected patients, by means of a study under real-life routine conditions in a mycobacteriology laboratory.

## Methods

This was a study of diagnostic accuracy, conducted under routine conditions at the bacteriology laboratory of the Evandro Chagas Clinical Research Institute, which is a referral center for the treatment of infectious diseases, located in the city of Rio de Janeiro, Brazil. All respiratory samples provided by HIV-infected patients suspected of having pulmonary tuberculosis and sent to the laboratory between January of 2008 and June of 2009 were included in the study. All samples collected from the same patient subsequent to the first sample were excluded from the study. Respiratory samples included sputum, induced sputum, and bronchoalveolar lavage samples.

**Figure 1:**
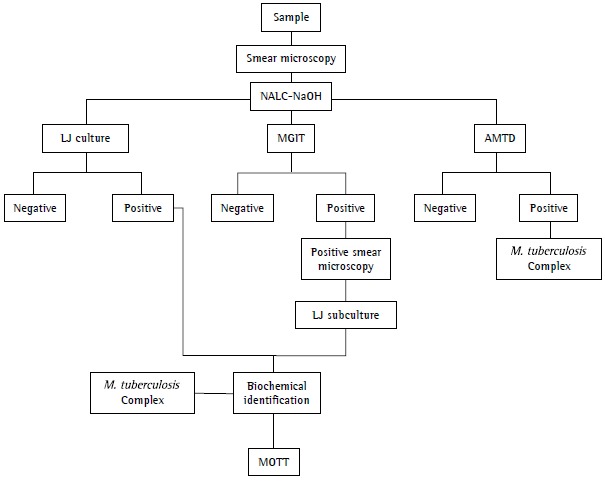
Sample processing flowchart. NALC-NaOH: N-acetyl-L-cysteine-sodium hydroxide; LJ: Löwenstein-Jensen; MGIT: Mycobacteria Growth Indicator Tube; AMTD: amplified Mycobacterium tuberculosis direct (test); and MOTT: mycobacteria other than tuberculosis

The clinical specimens were processed as shown in Figure 1. The samples were analyzed by smear microscopy, LJ culture, and the BACTEC Mycobacteria Growth Indicator Tube 960 (BACTEC MGIT 960) system (Becton Dickinson, Sparks, MD, USA). Smear microscopy was performed on the same day the clinical specimen was received at the laboratory. In contrast, cultures were performed over the course of 2 days at most. The samples showing growth on LJ medium, through culture or through subculture of positive BACTEC MGIT 960 cultures, were sent for biochemical identification of *M. tuberculosis* complex (detection of niacin production, nitrate reduction, and thermal inactivation of catalase).^(^ ^10^ ^)^ In the present study, cultures that produced niacin, reduced nitrate to nitrite, and showed inactivation of catalase at 68ºC were identified as positive for *M. tuberculosis* complex. Different results from those described above were analyzed and defined as positive for mycobacteria other than tuberculosis (MOTT). Part of the pellet obtained from decontamination of the samples was sent for AMTD tests and subsequent interpretation of results and for incubation in the BACTEC MGIT 960 system. Both methods were carried out as described by the respective manufacturers.^(^ ^11^ ^,^ ^12^ ^)^ A positive result was defined as the presence of *M. tuberculosis* complex in the sample, and a negative result was defined as the absence of *M. tuberculosis* complex. The AMTD test was performed weekly, and biochemical identification was obtained in the same week the cultures or subcultures yielded positive results. All collaborators who performed the tests mentioned above are regularly trained and evaluated on these procedures. There was no blinding of the collaborators, since they were performing routine tests.

The outcomes of interest were sensitivity, specificity, positive predictive value (PPV), negative predictive value (NPV), accuracy, likelihood ratio (LR), and respective 95% CIs. These measures were calculated using the Statistical Package for the Social Sciences, version 17.0 (SPSS, Chicago, IL, USA) and WINPEPI, version 11.15 (http://www.brixtonhealth.com/pepi4windows.html).

The present study was approved by the Research Ethics Committee of the Evandro Chagas Clinical Research Institute (Protocol no. 0002.0.009.000-11) and was developed in accordance with the recommendations of the Standards for Reporting Diagnostic Accuracy.^(^ ^13^ ^)^

A letter about the present study has been published.^(^ ^14^ ^)^

## Results

**Figure 2:**
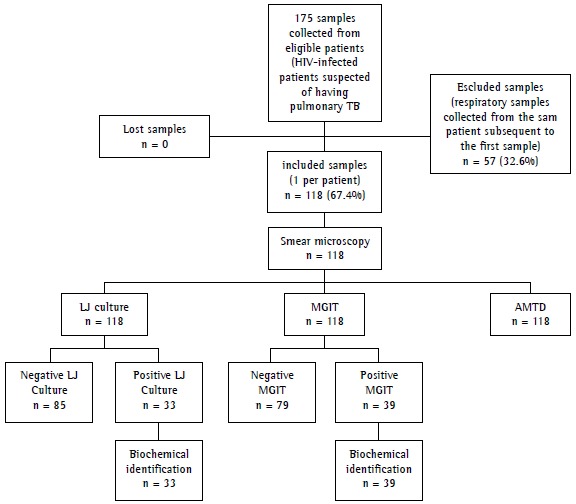
Study design diagram. TB: tuberculosis; LJ: Löwenstein-Jensen; MGIT: Mycobacteria Growth Indicator Tube; and AMTD: amplified Mycobacterium tuberculosis direct (test).

Of the 175 samples eligible for the study, 57 were excluded because they were subsequent samples from the same patient. Therefore, we analyzed the first respiratory samples collected from 118 patients, of whom 88 (74.4%) were male. The mean age was 36.6 ± 10.6 years. The results of all tests performed were conclusive. Figure 2 shows the study processing flowchart.

Of the 118 samples analyzed, 16 (13.6%) had positive results by smear microscopy. Of those 118 samples, 33 (27.9%) were positive by LJ culture, 1 of which was identified as MOTT, whereas (33.1%) were positive by the BACTEC MGIT 960 system, 3 of which were identified as MOTT and 1 of which was identified as *Rhodococcus *spp. The AMTD test detected 37 positive samples (31.4%) for *M. tuberculosis* complex. The isolated MOTT and *Rhodococcus *spp*.* strains were excluded from the main analysis because they are not targeted by the method under analysis.

After exclusion of those 5 samples, the comparison of the AMTD test results with those obtained by LJ culture showed that there were four false-negative results and nine false-positive results, whereas the comparison of the AMTD test results with those obtained by the BACTEC MGIT 960 system showed that there were six false-positive results and four false-negative results. Table 1 shows the diagnostic accuracy of the AMTD test in comparison with LJ culture and with the BACTEC MGIT 960 system.

**Table 1:** Accuracy of the amplified Mycobacterium tuberculosis direct test relative to culture on Löwenstein-Jensen medium and to the BACTEC Mycobacteria Growth Indicator Tube 960 system.a

Variable	AMTD vs*. *LJ	AMTD vs. MGIT
Sensitivity	87.5 (71.0-96.5)	88.6 (73.3-96.8)
Specificity	89.4 (80.8-95.0)	92.4 (84.2-97.2)
Positive predictive value	75.7 (58.8-88.2)	83.8 (68.0-93.8)
Negative predictive value	95.0 (87.7-98.6)	94.8 (87.2-98.6)
Positive likelihood ratio	8.25 (4.39-15.54)	11.66 (5.35-25.40)
Negative likelihood ratio	0.14 (0.06-0.35)	0.12 (0.05-0.31)
Accuracy	88.9 (81.7-93.9)	91.2 (84.5-95.7)

AMTD : amplified *Mycobacterium tuberculosis *direct (test)

LJ : Löwenstein-Jensen

MGIT : Mycobacteria Growth Indicator Tube

a Values expressed as % (95% CI).

In comparison with the BACTEC MGIT 960 system, the AMTD test had a sensitivity, specificity, PPV, and NPP of 88.6%, 92.4%, 83.8%, and 94.8%, respectively, whereas, in comparison with LJ culture, it showed values of 87.5%, 89.4%, 75.7%, and 95.0%, respectively. These results, together with their 95% CIs, the LRs, and the accuracy values, are shown in Table 1.

The same parameters were calculated for a subgroup of smear microscopy-negative samples, although that was not part of the initial analysis plan. The following results were obtained: sensitivity, 70.8% (95% CI: 48.6-87.3); specificity, 94.8% (95% CI: 87.2-98.6); PPV, 81.0% (95% CI: 58.1-94.6); and NPV, 91.3% (95% CI: 82.8-96.4).

## Discussion

In our study, regardless of the smear microscopy results, the AMTD test results showed sensitivity and specificity comparable to those in the literature. A systematic review of 125 studies not exclusively of patients with paucibacillary disease estimated a sensitivity of 85% and a specificity of 96.8% for commercial nucleic-acid amplification tests.^(^ ^15^ ^)^ A study not exclusively of patients with paucibacillary disease that compared the AMTD test with GeneXpert found a sensitivity of 96.8% and a specificity of 91.2% for the AMTD test. ^(^ ^16^ ^)^ It is possible that the difference observed relative to the values estimated in our study is due to the sample composition: whereas the eligibility criteria of the aforementioned study were too restrictive, our patients were selected only because they were seropositive for HIV.

The observed discrepant results, i.e., results that were positive by the AMTD test and negative by culture, may be due to laboratory contamination or to characteristics of the method used. The AMTD test can detect non-viable or dead bacilli, which are hardly to grow in culture. The opposite, i.e., results that were negative by the AMTD test and positive by culture, may indicate the presence of inhibitory substances, which were not examined in the present study.

A feature of nucleic-acid amplification tests is that sensitivity is compromised at the expense of specificity.^(^ ^15^ ^)^ Other factors that contribute to decreased sensitivity are poor, paucibacillary, or negative samples (in HIV-infected patients) and the presence of inhibitory substances.

The present study showed that, in tuberculosis/HIV-infected patients, the AMTD test was able to detect *M. tuberculosis* complex in a greater number of samples than culture. However, culture is not 100% sensitive and can yield false-negative results, such as when samples contain dead bacilli, non-viable bacilli (because of decontamination of samples), or less than the minimum detectable amount for culture (approximately 10^2 ^bacilli/mL). Therefore, the study results may have been influenced by the chosen reference test.

Although it was not the purpose of our study, analysis of the results of direct examination showed that only one smear microscopy-positive sample was not detected by the AMTD test, possibly because of inhibitors, given that *M. tuberculosis* was isolated by the two culture methods. Smear microscopy did not detect AFB in approximately 21% of the samples in which the AMTD test was positive. This shows the weakness of smear microscopy in detecting mycobacteria in patients who are seropositive for HIV. Several factors, such as the expertise of the technician; the quality of the sample, which needs to contain between 5,000 and 10,000 bacilli/mL in order to prevent false-negative results^(^ ^17^ ^,^ ^18^ ^)^; and particular conditions, such as HIV co-infection,^(^ ^18^ ^-^ ^20^ ^)^ directly influence smear microscopy results. However, smear microscopy remains an important tool for resource-poor countries, since it is the most rapid and inexpensive method available in all countries.

Studies have reported that the sensitivity of the AMTD test varies depending of the prevalence of HIV. However, they have shown the effectiveness of the method in identifying strains in smear microscopy-negative samples.^(^ ^21^ ^-^ ^23^ ^)^

The AMTD test is approved for use in respiratory samples regardless of smear microscopy results. It provides the greatest benefit to patients when used in smear microscopy-negative samples, given that it enables early diagnosis and the initiation of specific treatment. In our study, according to the reference method used, we obtained sensitivity and specificity similar to that reported by other studies not exclusively of HIV-infected patients.^(^ ^24^ ^-^ ^28^ ^)^

The main advantage of the routine use of nucleic-acid amplification tests in laboratories is the speed at which results are obtained, enabling early intervention when necessary. However, these tests should not replace culture, since they are able to detect non-viable microorganisms. For the same reason, they are also not useful for monitoring treatment, given that they provide non-quantitative results, which should be interpreted together with results of the conventional tests and with clinical data. However, they are useful in distinguishing between *M. tuberculosis* and MOTT, becoming an important tool in patients with heavy MOTT colonization/MOTT disease, as is the case of HIV-infected patients.

In conclusion, the AMTD test showed good sensitivity and specificity in the population studied, enabling the laboratory detection of *M. tuberculosis* complex in paucibacillary respiratory specimens.
