# Supplementary material for: Use of amplified Mycobacterium tuberculosis direct test in respiratory samples from HIV-infected patients in Brazil
Source: J Bras Pneumol. 2014 Mar-Apr;40(2):148–54. doi: 10.1590/S1806-37132014000200008 (PMC4083646; doi:10.1590/S1806-37132014000200008)
Supplement: Translation

# Translation: Utilização do *amplified Mycobacterium tuberculosis direct test* em amostras respiratórias de pacientes HIV positivos no Brasil*

**Authors:** Leonardo Bruno Paz Ferreira Barreto, Maria Cristina da Silva Lourenço, Valéria Cavalcanti Rolla, Valdiléia Gonçalves Veloso, Gisele Huf

**Affiliations:** Fundação Oswaldo Cruz, Rio de Janeiro (RJ) Brasil; Fundação Oswaldo Cruz, Rio de Janeiro (RJ) Brasil; Fundação Oswaldo Cruz, Rio de Janeiro (RJ) Brasil; Fundação Oswaldo Cruz, Rio de Janeiro (RJ) Brasil; Fundação Oswaldo Cruz, Rio de Janeiro (RJ) Brasil

**Keywords:** Técnicas de diagnóstico molecular, Tuberculose, HIV, Técnicas de sonda molecular

## Abstract

**OBJETIVO:**

Comparar a acurácia do teste *amplified Mycobacterium tuberculosis direct *(AMTD) com métodos de referência para o diagnóstico laboratorial de tuberculose em pacientes HIV positivos.

**MÉTODOS:**

Estudo de acurácia diagnóstica comparando os resultados do teste AMTD com os de cultura em Löwenstein-Jensen (LJ) e de BACTEC *Mycobacteria Growth Indicator Tube* 960 (sistema BACTEC MGIT 960) em amostras respiratórias analisadas no Laboratório de Bacteriologia e Bioensaios do Instituto de Pesquisa Clínica Evandro Chagas da Fundação Oswaldo Cruz, no Rio de Janeiro (RJ).

**RESULTADOS:**

Foram analisadas amostras respiratórias de 118 pacientes, dos quais 88 (74,4%) eram do sexo masculino. A média de idade foi de 36,6 ± 10,6 anos. O complexo *M. tuberculosis* foi identificado em 31,0%, 29,7% e 27,1% das amostras através do teste AMTD, sistema BACTEC MGIT 960 e LJ, respectivamente. Na comparação com a cultura em LJ, o teste AMTD apresentou sensibilidade, especificidade, valor preditivo positivo e valor preditivo negativo de 87,5%, 89,4%, 75,7% e 95,0%, respectivamente, enquanto na comparação com o sistema BACTEC MGIT 960, os valores foram de 88,6%, 92,4%, 83,8% e 94,8%, respectivamente.

**CONCLUSÕES:**

O teste AMTD mostrou boa sensibilidade e especificidade na população estudada, possibilitando a detecção laboratorial do complexo *M. tuberculosis* em espécimes respiratórios paucibacilares.

## Introdução

Apesar de mais de um século da descoberta do agente etiológico da tuberculose, *Mycobacterium tuberculosis*, ela continua sendo um problema de saúde pública de âmbito mundial. Cada indivíduo que apresenta tuberculose em atividade infecta de 10 a 15 pessoas em um ano.^(^ ^1^ ^)^ Estima-se que a cada 10 pessoas que entram em contato com o bacilo da tuberculose pelo menos uma desenvolva a doença e que, nos pacientes HIV positivos, esse risco aumente de 20 a 40 vezes.^(^ ^2^ ^)^ Estudos que avaliaram a sobrevida em indivíduos com coinfecção tuberculose/HIV demonstraram um maior risco de morte nesses pacientes do que naqueles com HIV sem tuberculose.^(^ ^3^ ^-^ ^6^ ^)^

O método de isolamento considerado o padrão ouro é a cultura para micobactérias em meio sólido de Löwenstein-Jensen (LJ).^(^ ^7^ ^)^ Esse método tem como limitação o longo tempo de incubação (2-8 semanas), mas é utilizado pela maioria dos países em desenvolvimento devido ao seu baixo custo. Técnicas, como a amplificação de ácidos nucleicos e sistemas automatizados utilizando culturas em meio líquido, possuem elevado custo e dependem de ferramentas sofisticadas, o que as inviabilizam para o uso rotineiro em países pobres.

Na última década, os exames laboratoriais para detecção do *M. tuberculosis *evoluíram sensivelmente.^(^ ^8^ ^)^ Hoje podemos contar com novas metodologias, tais como GeneXpert (Cepheid, Sunnyvale, CA, EUA), capaz de gerar um resultado em 2 h com a detecção do complexo *M. tuberculosis *e se as cepas apresentam resistência a rifampicina; no entanto, essa ainda apresenta um alto custo e começa a ser utilizada e validada em nosso meio. O teste *amplified Mycobacterium tuberculosis direct *(AMTD; Gen-Probe, San Diego, CA, EUA) é capaz de detectar o RNAr do complexo *M. tuberculosis* em aproximadamente 3 h. Esse método foi aprovado pelo *Food and Drug Administration* para amostras respiratórias com baciloscopia positiva desde 1995 e, após um aprimoramento em 1999, o teste foi aprovado para a aplicação em amostras com baciloscopia negativa.^(^ ^9^ ^)^ Ainda é necessário um melhor entendimento do desempenho desse teste para a clientela paucibacilar, como pacientes HIV positivos em nosso meio, já que a qualidade de suas amostras habitualmente dificulta o diagnóstico laboratorial até para os métodos considerados como padrão ouro, como a cultura em meio líquido. O presente estudo teve como objetivo comparar a acurácia diagnóstica do teste AMTD com outros métodos de cultura em amostras respiratórias de pacientes HIV positivos, através de um estudo em condições reais da prática diária de um laboratório de micobactérias.

## Métodos

Este é um estudo de acurácia diagnóstica, realizado nas condições de rotina do laboratório de bacteriologia do Instituto de Pesquisa Clínica Evandro Chagas, referência de tratamento em doenças infecto-contagiosas, localizado na cidade do Rio de Janeiro (RJ). Foram incluídas no estudo todas as amostras respiratórias fornecidas por pacientes HIV positivos com suspeita de tuberculose pulmonar e encaminhadas para o laboratório no período entre janeiro de 2008 e junho de 2009. Foram excluídas do estudo todas as amostras coletadas subsequentemente à primeira, fornecidas pelos pacientes incluídos no período do estudo. Foram consideradas como amostras respiratórias amostras de escarro, escarro induzido e lavado broncoalveolar.

O processamento dos espécimes clínicos foi realizado de acordo com a Figura 1. As amostras foram submetidas a baciloscopia e cultura em LJ e sistema BACTEC *Mycobacteria Growth Indicator Tube* 960 (BACTEC MGIT 960; Becton Dickinson, Sparks, MD, EUA). A baciloscopia foi realizada no mesmo dia do recebimento do espécime clínico no laboratório. Já as culturas foram executadas ao longo de, no máximo, 2 dias. As amostras que apresentaram crescimento em LJ, a partir da cultura ou do subcultivo proveniente do sistema BACTEC MGIT 960 positivo, foram encaminhadas para a identificação bioquímica do complexo *M. tuberculosis* (detecção da produção de niacina, redução do nitrato e termoinativação da catalase). ^(^ ^10^ ^)^ No presente estudo, foram identificadas como complexo *M. tuberculosis* as culturas que produziram niacina, reduziram o nitrato a nitrito e apresentaram inativação de catalase a 68ºC. Resultados diferentes dos citados anteriormente foram analisados e considerados como *mycobacteria other than tuberculosis *(MOTT, micobactérias que não tuberculose). Parte do sedimento obtido na descontaminação da amostra foi encaminhada para a execução e interpretação do teste AMTD e incubação no sistema BACTEC MGIT 960. Ambas as metodologias foram processadas conforme descrito pelos respectivos fabricantes. ^(^ ^11^ ^,^ ^12^ ^)^ O resultado positivo foi interpretado como presença do complexo *M. tuberculosis*, e o negativo, como sua ausência na amostra. O teste AMTD foi realizado semanalmente, e as identificações foram obtidas nas mesmas semanas em que as culturas ou subcultivos apresentaram resultados positivos. Todos os colaboradores que executaram os testes citados são treinados e avaliados periodicamente no cumprimento dos respectivos procedimentos. Não houve cegamento dos mesmos, já que se tratava da execução de exames de rotina.

**Figura 1:** Fluxo de processamento das amostras. NALC-NaOH: N-acetil-L-cisteína-hidróxido de sódio; LJ: Löwenstein-Jensen; MGIT: Mycobacteria Growth Indicator Tube; AMTD: teste amplified Mycobacterium tuberculosis direct; e MOTT: mycobacteria other than tuberculosis (micobactérias que não tuberculose).

Os desfechos de interesse foram os valores de sensibilidade, especificidade, valor preditivo positivo (VPP), valor preditivo negativo (VPN), acurácia, razão de verossimilhança (RV) e respectivos IC95%. Essas medidas foram calculadas utilizando-se os programas *Statistical Package for the Social Sciences*, versão 17.0 (SPSS, Chicago, IL, EUA) e WINPEPI, versão 11.15 (http://www.brixtonhealth.com/pepi4windows.html).

O presente estudo foi aprovado pelo Comitê de Ética e Pesquisa do Instituto de Pesquisa Clínica Evandro Chagas sob o nº 0002.0.009.000-11 e foi desenvolvido segundo as recomendações do *Standards for Reporting Diagnostic Accuracy*.^(^ ^13^ ^)^

Uma correspondência referente ao presente estudo foi publicada.^(^ ^14^ ^)^

## Resultados

Das 175 amostras elegíveis para o estudo, 57 foram excluídas, pois se tratavam de amostras subsequentes do mesmo paciente. Portanto, foram avaliadas as primeiras amostras respiratórias de 118 pacientes, dos quais 88 (74,4%) eram do sexo masculino. A média de idade foi de 36,6 ± 10,6 anos. Todos os procedimentos realizados obtiveram resultados conclusivos. A Figura 2 mostra o diagrama do estudo.

**Figura 2:** Diagrama do estudo. TB: tuberculose; LJ: Löwenstein-Jensen; MGIT: Mycobacteria Growth Indicator Tube; e AMTD: teste amplified Mycobacterium tuberculosis direct.

Das 118 amostras analisadas, 16 (13,6%) apresentaram baciloscopia positiva. Das 118 amostras, 33 (27,9%) foram positivas pelo método LJ, sendo 1 identificada como MOTT, enquanto 39 (33,1%) foram positivas pelo método BACTEC MGIT 960, sendo 3 identificadas como MOTT e 1 como *Rhodococcus *spp. O teste AMTD detectou 37 amostras positivas (31,4%) para o complexo *M. tuberculosis*. As cepas MOTT e *Rhodococcus *spp*.* isoladas foram excluídas da análise principal por não serem alvo da metodologia avaliada.

Após a exclusão daquelas 5 amostras, houve quatro resultados falso-negativos e nove resultados falso-positivos na comparação com o LJ, enquanto houve seis resultados falso-positivos e quatro resultados falso-negativos na comparação com o BACTEC MGIT 960. A Tabela 1 mostra os valores de acurácia diagnóstica do teste AMTD quando comparado aos métodos LJ e BACTEC MGIT 960.

**Tabela 1:** Acurácia do teste amplified Mycobacterium tuberculosis direct em relação aos métodos de cultura em Löwenstein-Jensen e BACTEC Mycobacteria Growth Indicator Tube 960.a

Variáveis	AMTD vs*. *LJ	AMTD vs. MGIT
Sensibilidade	87,5 (71,0-96,5)	88,6 (73,3-96,8)
Especificidade	89,4 (80,8-95,0)	92,4 (84,2-97,2)
Valor preditivo positivo	75,7 (58,8-88,2)	83,8 (68,0-93,8)
Valor preditivo negativo	95,0 (87,7-98,6)	94,8 (87,2-98,6)
Razão de verossimilhança positiva	8,25 (4,39-15,54)	11,66 (5,35-25,40)
Razão de verossimilhança negativa	0,14 (0,06-0,35)	0,12 (0,05-0,31)
Acurácia	88,9 (81,7-93,9)	91,2 (84,5-95,7)

AMTD : *amplified Mycobacterium tuberculosis direct test*

LJ : Löwenstein-Jensen

MGIT : *Mycobacteria Growth Indicator Tube*

a Valores expressos em % (IC95%).

Na comparação do teste AMTD com BACTEC MGIT 960, os resultados de sensibilidade, especificidade, VPP e VPN foram de 88,6%, 92,4%, 83,8% e 94,8%, respectivamente. Na comparação do teste AMTD com LJ, esses foram de 87,5%, 89,4%, 75,7% e 95,0%, respectivamente. Tais resultados, juntamente com os IC95%, RV e acurácia, são apresentados na Tabela 1.

Embora não estivesse previsto no plano de análise inicial, foram calculados os mesmos parâmetros para um subgrupo de amostras com baciloscopia negativa. Foram obtidos os seguintes resultados: sensibilidade, 70,8% (IC95%: 48,6-87,3); especificidade, 94,8% (IC95%: 87,2-98,6); VPP, 81,0% (IC95%: 58,1-94,6); e VPN, 91,3% (IC95%: 82,8-96,4).

## Discussão

Em nosso estudo, independentemente do resultado de baciloscopia das amostras, o teste AMTD apresentou sensibilidade e especificidade comparáveis com dados da literatura. Uma revisão sistemática que incluiu 125 estudos em pacientes não exclusivamente paucibacilares estimou uma sensibilidade de 85% e uma especificidade de 96,8% para técnicas de amplificação de ácidos nucleicos comerciais.^(^ ^15^ ^)^ Um estudo comparando o teste AMTD e GeneXpert em pacientes não exclusivamente paucibacilares, obteve-se uma sensibilidade de 96,8% e uma especificidade de 91,2% para o teste AMTD.^(^ ^16^ ^)^ É possível que a diferença observada em relação aos valores estimados em nosso estudo possa se dever à composição da amostra: enquanto os critérios de elegibilidade foram muito restritivos no estudo acima citado, nossos pacientes eram selecionados apenas por serem soropositivos para o HIV.

Os resultados discordantes observados, positivo para o teste AMTD e negativo para cultura, podem se dever à contaminação laboratorial ou a características da metodologia utilizada. O teste AMTD pode detectar bacilos mortos ou não viáveis, que dificilmente cresceriam na cultura. O contrário, resultado negativo para o teste AMTD e positivo para cultura, pode indicar a presença de substâncias inibidoras, as quais não foram examinadas no presente estudo.

Uma característica das técnicas de amplificação de ácidos nucleicos é o comprometimento da sensibilidade em detrimento da especificidade.^(^ ^15^ ^)^ Outros fatores que contribuem para a diminuição da sensibilidade são amostras pobres, paucibacilares ou negativas (em pacientes HIV) e a presença de substâncias inibidoras.

O presente estudo mostrou que o teste AMTD foi capaz de detectar um maior número de amostras positivas para o complexo *M. tuberculosis* do que as culturas na população tuberculose/HIV positiva. Entretanto, a cultura não é 100% sensível, podendo apresentar resultados falso-negativos, como, por exemplo, em casos de bacilos mortos, bacilos inviabilizados pela descontaminação da amostra, ou quantidade inferior ao mínimo para a detecção pela cultura (cerca de 10^2 ^bacilos/mL). Portanto, o resultado do estudo pode ter sido influenciado pelo teste de referência escolhido.

Embora não fosse a proposta do nosso estudo, ao analisar os resultados de exame direto, apenas uma amostra com baciloscopia positiva não foi detectada pelo teste AMTD, possivelmente por conta de inibidores, uma vez que foi isolado *M. tuberculosis* em ambos os métodos de cultura. A baciloscopia não detectou BAAR em aproximadamente 21% das amostras em que o teste AMTD foi positivo. Isso mostra a fragilidade da baciloscopia na detecção de micobactérias na população HIV soropositiva. Diversos fatores influenciam diretamente o resultado da baciloscopia, tais como a experiência do técnico; a qualidade da amostra, que necessita apresentar entre 5.000 e 10.000 bacilos/mL para evitar a ocorrência de resultados falso-negativos^(^ ^17^ ^,^ ^18^ ^)^; e condições peculiares, como a coinfecção pelo HIV.^(^ ^18^ ^-^ ^20^ ^)^ Entretanto, a baciloscopia ainda é uma ferramenta importante para países com poucos recursos, já que é o método mais rápido e mais barato disponível em todos os países.

Estudos reportaram uma sensibilidade variável do teste AMTD dependendo da prevalência de HIV. Entretanto, mostraram a competência do método na identificação das cepas em amostras com baciloscopia negativa.^(^ ^21^ ^-^ ^23^ ^)^

O teste AMTD está aprovado para uso em amostras respiratórias independentemente do resultado de baciloscopia. A situação em que se proporciona o maior benefício ao paciente é o uso dessa metodologia em amostras com baciloscopia negativa, uma vez que permite o diagnóstico precoce e a introdução do tratamento específico. Em nosso estudo, de acordo com o método de referência empregado, obtivemos sensibilidade e especificidade semelhantes aos resultados encontrados por outros autores em pacientes não exclusivamente HIV.^(^ ^24^ ^-^ ^28^ ^)^

A maior vantagem da utilização de uma técnica de amplificação de ácidos nucleicos na rotina laboratorial é a velocidade em que se obtém o resultado, possibilitando a rápida intervenção quando necessário. Entretanto, essas técnicas não devem substituir a cultura, já que são capazes de detectar o microrganismo não viável. Pela mesma razão, também não são úteis para o monitoramento do tratamento, uma vez que o resultado não é quantitativo, devendo ser interpretados em conjunto com os testes convencionais e dados clínicos. No entanto, eles são úteis na distinção entre *M. tuberculosis* e MOTT, tornando-se uma ferramenta importante em populações que possuam alta colonização/doença por MOTT, como é o caso dos pacientes HIV positivos.

Em conclusão, o teste AMTD mostrou boa sensibilidade e especificidade na população estudada, possibilitando a detecção laboratorial do complexo *M. tuberculosis* em espécimes respiratórios paucibacilares.
